# Effects of Baicalin on Alopecia and the Associated Mechanism

**DOI:** 10.1155/2022/3139123

**Published:** 2022-11-18

**Authors:** Liping Chen, Bo Fan, Huan Gu, Liuqing Yang, Xiaofang Li

**Affiliations:** ^1^School of Pharmacy, Chengdu University of Traditional Chinese Medicine, Chengdu, Sichuan 611137, China; ^2^School of Food and Bioengineering, Xihua University, Chengdu, Sichuan 610039, China

## Abstract

The aim of the present study was to explore the potential pharmacological mechanism of baicalin by combining network pharmacology prediction and the experimental verification of alopecia. Networks of baicalin-associated targets and alopecia-related genes were constructed using the STRING database. Potential targets and pathways associated with the therapeutic efficacy of baicalin were identified via enrichment analysis using Cytoscape and the database for annotation, visualization and integrated discovery (Metascape). The back hair of C57BL/6J mice was removed with depilatory cream to verify the therapeutic effect of baicalin. Human hair dermal papilla cells (HHDPCs) were used to explore the mechanism of action of baicalin. Network pharmacology analysis revealed that the potential targets of baicalin mainly include protein serine/threonine kinase, Src protein, epidermal growth factor receptor, and insulin-like growth factor 1 (IGF1), which were indicated to mediate neutrophil degranulation and regulation of cell-cell adhesion, vesicle lumen, cytoplasmic vesicle, membrane raft, and endopeptidase activity. Multiple pathways were identified, such as proteoglycans in cancer, PI3K/AKT, and forkhead box O signaling pathways. Following baicalin treatment for the experimental mice, the coverage, length, and weight of the hair increased in a baicalin dose-dependent manner. Moreover, the histological evaluation showed that the number of hair follicles increased after baicalin treatment and melanin formation were pronounced. In addition, baicalin induced an increase in the phosphorylated p-AKT, p-glycogen synthase kinase-3*β*, p-PI3K, TGF-*β*1, and vascular endothelial growth factor levels. Furthermore, the activation levels of key protein p-AKT were increased. Baicalin induced the proliferation of HHDPCs *in vitro* and significantly upregulated p-AKT, IGF1, and alkaline phosphatase. In conclusion, the present study revealed that the pharmacological mechanisms of baicalin in alopecia therapy were associated with the proliferation of DPCs, the activation of the AKT pathway, and the transmission of downstream signals, indicating that baicalin is a potential drug candidate for the clinical treatment of hair loss.

## 1. Introduction

Alopecia areata has a significant impact on the mental wellbeing of patients and their social interactions [1]. At present, the main methods of hair loss prevention and treatment are surgery and medication. Surgery is an invasive treatment option that may have complications, such as bleeding, infection, swelling, scar growth, and epidermoid cysts, in addition to being costly [2]. Drug therapy is used to prevent alopecia by promoting hair growth. Commonly used drugs include minoxidil, finasteride, RU58841, cyclosporine A, and FK50, but they are all associated with certain defects, such as an increased heart rate and arrhythmia [3, 4]. The duration of alopecia treatment is long and oral medications frequently cause severe liver and kidney burdens [3, 5]. Therefore, developing highly effective drugs with clear mechanisms of action and nontoxic side effects is of great social and economic importance for the prevention and treatment of alopecia.

At present, the treatment of hair loss with natural compounds such as glucosides is receiving increasing attention. An early study indicated that baicalin, an active monomer derived from traditional Chinese medicine (TCM), is able to promote the proliferation of human hair dermal papilla cells (HHDPCs) [6]. DPCs have been reported to be closely linked to hair growth. They are a type of dermal-derived stem cells with abundant sources and easy-to-obtain materials [7]. They maintain strong developmental plasticity and have highly specialized biological functions [8]. Baicalin belongs to the polyphenol flavonoids, which are widely distributed in TCM plants, such as *Scutellaria* and *Barbara* [9]. Flavonoids have been reported to be beneficial to human health and have health functions and may also be used as highly effective drugs in clinical applications [10]. Baicalin has a significant anti-inflammatory effect on skin inflammation and several other inflammatory diseases [11]. Baicalin may reduce liver damage, the content of alanine aminotransferase, and NF-*κ*B as well as the mRNA and protein levels of TNF-*α* and may protect mouse liver cells from lipopolysaccharide damage [12]. However, there are no studies on the treatment of alopecia by baicalin. Therefore, in the present study, network pharmacology was first applied to study the potential targets and pathways of baicalin for the treatment of alopecia.

Network pharmacology is a new discipline based on the theory of systems biology, which analyzes the network of biological systems and selects specific signal nodes for multitarget drug molecular design [13]. Systems biology, network biology, gene redundancy, and other principles were used to explain the process of disease development; it also uses a holistic view of network balance to understand the interaction between drugs and the body [14]. Network pharmacology has a high application in the medical treatment of complex diseases. Natural products are rich in structure and biological activity diversity and have numerous targets. A preliminary experimental study was performed on Chinese herbal *Scutellaria baicalensis* Georgi, *Rheum palmatum L.*, *Angelica sinensis*, *Isatis indigotica* Fort*.,* and *Fallopia multiflora (Thunb.)* Harald [15]. Baicalin is a monomeric compound, which is an important active ingredient of the formula. Therefore, in the present study, network pharmacology was used to study the mechanism of the effect of baicalin on alopecia *in vivo* and *in vitro*, providing a theoretical basis for the treatment of clinical alopecia and new ideas for the study of active ingredients of TCM.

## 2. Materials and Methods

### 2.1. Network Pharmacology Analysis

The active compound of baicalin was retrieved from five different TCM databases (TCMSP-https://old.tcmsp-e.com/tcmsp.php, TCMID-https://ngdc.cncb.ac.cn/databasecommons/database/id/437, TCM-PTD-http://tcm.zju.edu.cn/ptd/, and HIT-http://lifecenter.sgst.cn/hit/). PubChem (https://pubchem.ncbi.nlm.nih.gov/) and ChemDraw software 17 (CambridgeSoft) were used to obtain SDF-format files for baicalin. SwissTargetPrediction (http://www.swisstargetprediction.ch/) was used to predict the potential targets of baicalin and screen the targets with higher scores (≥0.2). Next, based on the GeneCards (https://www.genecards.org/) database, “alopecia” was used as the keyword to search for alopecia disease-related targets (screening criteria relevance score ≥ 40). Disease and compound targets were uploaded to calculate and custom Venn diagrams were drawn (http://bioinformatics.psb.ugent.be/webtools/Venn/) to obtain a common set of targets and initially explore the potential role of baicalin in the treatment of alopecia.

The STRING database (https://string-db.org/) was used to perform protein-protein interaction (PPI) analysis on intersection targets with *Homo sapiens* selected and medium confidence (≥0.7). The interaction information between proteins was used to construct a PPI network diagram. Cytoscape 3.8.2 software (http://manual.cytoscape.org/en/stable/) was used to perform a topological analysis on the PPI network graph. The analysis focused on degree (≥0.5) and betweenness centrality (≥0.002). Next, using the Metascape database (http://metascape.org/gp/index.html), Gene Ontology (GO) enrichment analysis of molecular function (MF), cellular component (CC), and biological process (BP) were performed on intersection targets. Finally, Kyoto Encyclopedia of Genes and Genomes (KEGG) signaling pathway enrichment analysis was carried out using the Metascape database. Entries with *P* ≤ 0.05 were screened out, and a bubble chart was generated for statistical analysis of these items. GO enrichment and KEGG analyses were performed to obtain the pathways involved in the important molecular mechanism of baicalin in the treatment of alopecia.

### 2.2. Cell Culture

Primary culture HHDPCs were purchased from Shanghai Xuanyuan Biotechnology Co., Ltd. (https://china.guidechem.com/trade/pdetail15970820.html/). The use of primary cells in this study was approved by the Medical Ethics Committee of the Affiliated Hospital of Chengdu University of TCM (Chengdu, China). HHDPCs were cultured in DMEM (BI, Israel) containing 10% FBS (BI, Israel) in a cell incubator with 5% CO_2_ at 37°C. When they had grown to 80-90% confluence, 20% of the cells were subcultured. LY294002 (MedChemExpress, US) was dissolved in dimethyl sulfoxide (DMSO, Yeasen, China), stored in a -80°C freezer and diluted with prewarmed culture medium to appropriate concentrations 10 min prior to drug treatment (DMSO concentration below 0.01%).

### 2.3. Animals and Grouping

Specific pathogen free-grade C57BL/6 female mice (body weight, 20 ± 2 g; age, 5 weeks) were reared in a separate cage (1 mouse per cage). The mice were given free access to water and food at 20-26°C and 40%-70% humidity, alternating between light and dark for 12 h/12 h. The mice were provided by the Experimental Animal Center of Xihua University [SYXK (Chuan) 2014-124], and all animals were given free access to water and food. In the present study, all experimental procedures were performed following the guidelines of the Animal Experimental Committee and the Ethics Committee of Chengdu University of TCM (IRB number: 2019-28), animal ethics approval for the present study was received on March 20, 2019, and the number of animals used was kept to a minimum (*n* = 6, 6 mice per group). Following the completion of the experiments, the animals were intraperitoneally injected with 1% sodium pentobarbital (50 mg/kg) and then sacrificed by CO_2_ inhalation. The CO_2_ emission was 20% of the chamber volume per min. Subsequently, the euthanasia chamber was filled with CO_2_ until it reached the maximum concentration of 99.9% (5 min): mouse chest no fluctuation more than 5 min, determine the death of mice, and tissue samples were then obtained.

All mice were acclimatized for 1 week prior to the experiment. Depilatory cream (Veet, China) was used to remove hair from a 3~4 cm^2^ area on the back of the mice (*n* = 6). Following hair removal, the mice were allowed to recover for 2 days. Injured mice (skin damage and bleeding) were excluded from the experiment, and mice with a similar appearance of the skin were used for the experiments. When the skin of the mice was pink and undamaged and the hair follicles of the mice were in the resting state, the next experiments were performed [16, 17]. The 30 experimental mice were randomly divided into the control group (depilated area of mice were coated with the drug vehicle of the experimental group and the same dose was used at the same time-points), positive control drug (minoxidil 10 mg/ml; MedChemExpress), and 10, 30, and 90 mg/ml baicalin (MedChemExpress) treatment groups. The drug solvent was composed of glycerol, anhydrous ethanol, and sterile water at a ratio of 3 : 10 : 11. The drug solution (minoxidil and baicalin) was applied twice a day until hair grew out of the area on the back of the mice from which hair had been removed.

### 2.4. Evaluation of Hair Growth Cycle

The skin color and hair growth of the hair removal area of each group were observed daily. The time when the skin color of each mouse changed from pink to black and the time when the hair started to grow were recorded to evaluate the effect of the drug on the hair growth cycle of the mice. Photographic images were acquired in each group on days 0, 7, 14, and 28, and hair growth was recorded.

### 2.5. Determination of the Hair Length and Weight

Changes in the skin and hair of mice were observed daily. On days 14 and 28, 5-10 hairs were randomly selected from the same part of each mouse and measured under the microscope. The length of the hair was recorded at the furthest distance between the two ends of the hair to calculate the average length. The data were analyzed using GraphPad Prism 8.0 (GraphPad Software, Inc.). After the end of the experiment, the animals were injected with 1% sodium pentobarbital (50 mg/kg) and sacrificed by carbon dioxide inhalation. From each group of mice, the same location of the back skin area was selected. 0.4 cm^2^ areas were obtained, and the gross weight was calculated.

### 2.6. Hematoxylin and Eosin (H&E) Staining and Evaluation

Following baicalin or control treatments, experimental mouse skin tissue was collected and fixed in 4% paraformaldehyde for 24 h for dehydration. Next, 5-*μ*m-thick sections were prepared. H&E staining (C0105S; Beyotime Institute of Biotechnology) was used to observe the difference in the number and morphology of hair follicles under a microscope.

### 2.7. MTT Assay

Cells in the logarithmic growth phase were inoculated in a 96-well cell culture plate at a seeding density of 10^4^ cells/well and a liquid volume of 200 *μ*l/well. When the cells had adhered to the bottom of the wells, the culture medium was aspirated and 200 *μ*l of baicalin culture medium with different concentration gradients (0-1 *μ*g/ml) was added, followed by incubation for 72 h. Next, 20 *μ*l of 5 g/l MTT (T100896; Shanghai Aladdin Biochemical Technology Co., Ltd.) was added to each well for a 4 h incubation at 37°C in a CO_2_ incubator. Finally, the culture medium was discarded, 200 *μ*l DMSO was added to each well, the culture plate well was shaken, and the absorbance of each well was measured at 490 nm with a microplate reader.

### 2.8. Reverse Transcription-Quantitative PCR (RT-qPCR)

RNA extraction was performed using the RNATRIzol extraction kit (RR9109; Takara Bio, JAP), according to the manufacturer's protocol. RT for cDNA synthesis was performed as follows: OligodT (RR047A; Takara Bio, JAP) was used as a primer to reverse-transcribe 1 *μ*g RNA into cDNA. The reaction system (20 *μ*l) consisted of 5X M-MLV buffer 4 *μ*l, dNTP 10 mmol, 20 U RNase, 100 U M-MLV, 20 pmol oligo (dT), and 1 *μ*g RNA. The reaction conditions were as follows: 20°C for 10 min, 42°C for 60 min, and 95°C for 5 min. PCR amplification was performed using 2 *μ*l RT products with SYBR Green (RR820A; Takara Bio, JAP) with *β*-actin as an internal reference. QuantStudio 5 (Thermo Fisher Scientific, USA) real-time fluorescence quantitative PCR instrument is used for the detection of mRNA expression. The PCR cycling conditions were as follows: 95°C for 5 min, 35 cycles of 94°C for 60 sec, 57°C for 45 sec, and 72°C for 60 sec and a final hold at 72°C for 10 min. Gene expression relative to *β*-actin was calculated using the 2^-∆∆Ct^ method. Primer sequences are listed in Table 1.

### 2.9. Western Blot Analysis

Western blot analysis was used to detect proteins involved in hair growth. RIPA lysis buffer was used for extraction of proteins from tissues. The BCA method was used to determine the protein concentration, and 40 *μ*g total protein was loaded for 10% SDS-PAGE, followed by transfer to a PVDF membrane (Millipore, USA) and 5% skim milk powder blocking for 1 h (Wandashan, China). Primary antibodies AKT (1 : 1000, ab283852, Abcam, UK), phosphorylated p-AKT (1 : 500, ab8805, Abcam, UK), insulin-like growth factor-1 (IGF1, 1 : 1000, ab182408, Abcam, UK), alkaline phosphatase (ALP, 1 : 1000, ab229126, Abcam, UK), and *β*-actin (1 : 7000, ab8227, Abcam, UK) were used for incubation overnight at 4°C. Incubation with the secondary antibody followed (IgG antibody 1: 5000; D190786, Shanghai Shenggong BBI Life) at 37°C for 1 h. Image-J software6.0 (National Institutes of Health) was used to analyze the protein bands in grayscale, and the gray value ratio of the target protein to the internal reference *β*-actin band was used as the relative expression level of the target protein.

### 2.10. Statistical Analysis

Values are expressed as the mean ± standard deviation. The data obtained in the experiments were all statistically analyzed using GraphPad Prism 8.0 software (GraphPad Software, Inc.). The Shapiro-Wilk test was used to test the normality of distribution of the data, suggesting that the data were normally distributed and consistent with homogeneity of variance. Comparison between groups was performed using one-way ANOVA with Tukey's post hoc test of means. *P* < 0.05 was considered to indicate a statistically significant difference.

## 3. Results

### 3.1. Network Pharmacology Analysis

**Figure 1:**
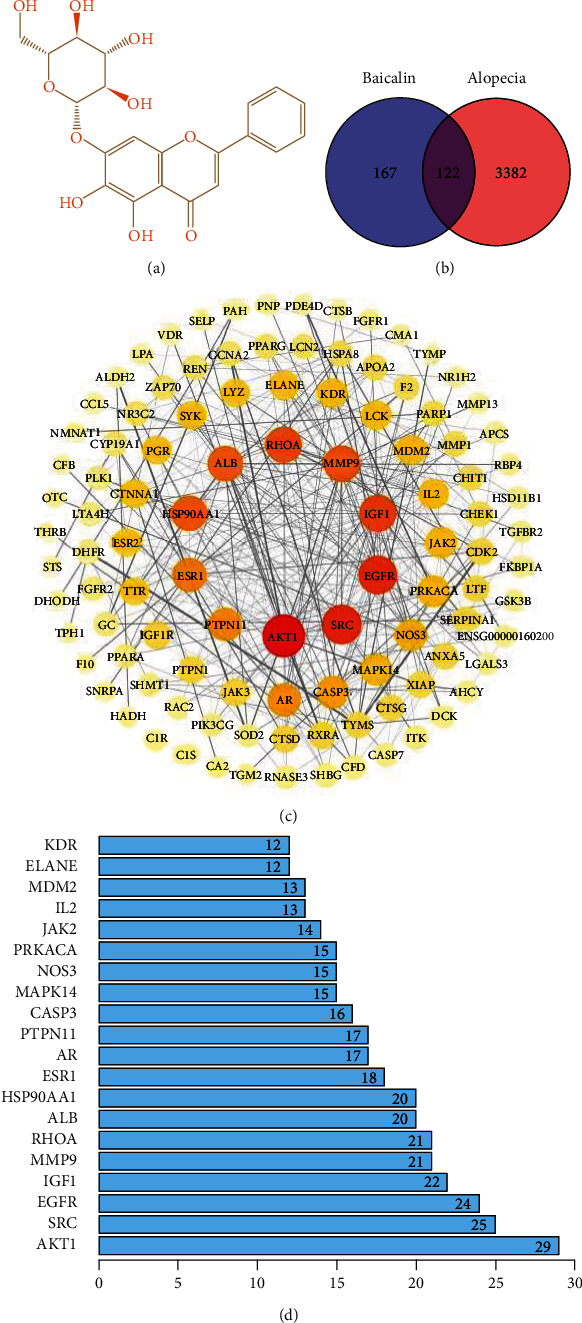
Targets of baicalin in the treatment of alopecia were explored. (a) Chemical structure of baicalin. (b) Venn diagram of targets of medicine and disease. (c) Protein-protein interaction network of intersection targets. (d) Key targets of baicalin in the treatment of alopecia.

Baicalein (chemical name, 7-O-*β*-D-glucoside) is a flavonoid glucoside (Figure 1(a)). A total of 289 target proteins were predicted for baicalin, 3,504 targets for alopecia diseases were obtained from GeneCards databases, and 122 identical targets for the compound and alopecia were identified. The intersection target PPI network was obtained from the STRING database (Figure 1(b)). The network had 121 nodes, 383 edges, and an average degree of 6.33. The larger the node, the darker the color and the greater the degree value. Core therapeutic targets include AKT1 and epidermal growth factor receptor (EGFR, Figure 1(c)). The thickness of the line (from thick to thin) indicated that the edge betweenness transitioned from large to small. The genes with scores greater than the average score were selected as key targets, a total of 106 key targets were screened out, and the first 20 targets were graphically presented (Figure 1(d)). Next, among the top 20 targets, PI3K, AKT, IGF1, ALP, GSK-3*β*, and TGF-*β*1 are the proteins we focus on.

### 3.2. Bioinformatics Analysis of Core Targets

**Figure 2:**
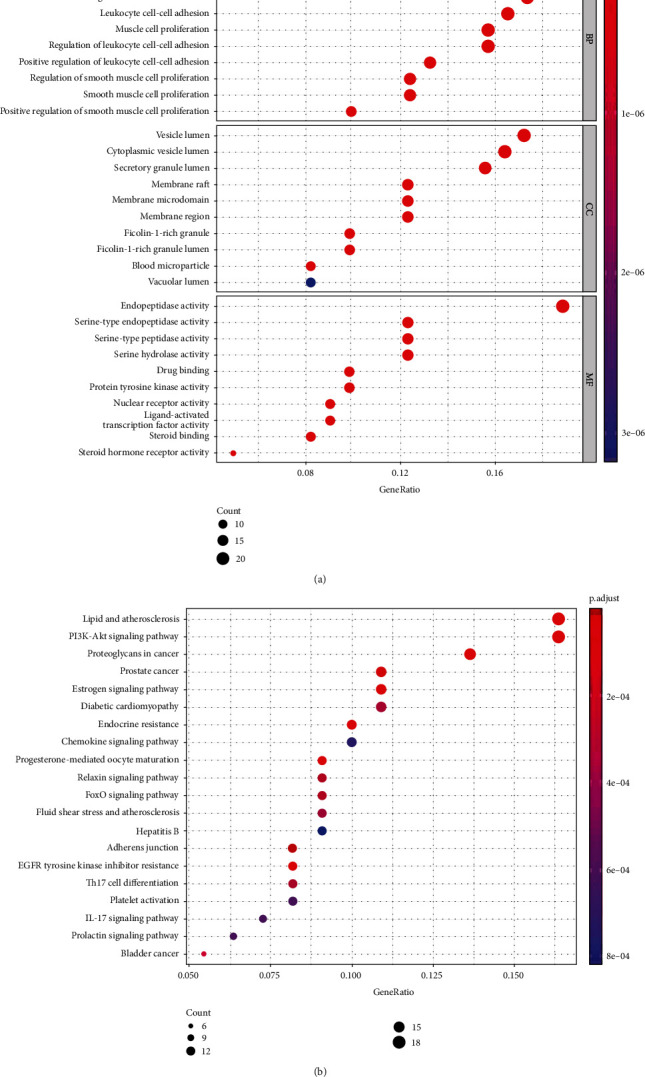
GO analysis of potential targets and significantly enriched KEGG pathways based on predicted targets. (a) GO enrichment analysis. (b) KEGG pathway enrichment. GO: gene ontology; KEGG: Kyoto Encyclopedia of Genes and Genomes. The *y*-axis represents the pathway, the *x*-axis represents the percentage of genes, the bubble area represents the number of pathway enrichment genes, and the bubble color represents the size of the *P* value.

GO enrichment analysis of 122 potential targets was performed through the Metascape database to obtain BP, CC, and MF terms for target genes, using *P* < 0.05 as the screening criterion. A total of 1,526 terms in the category BP, 37 in CC, and 112 in MF were obtained. The top 10 entries were selected to draw the bubble chart (Figure 2(a)). The BP terms at the intersection mainly involved neutrophil degranulation and regulation of cell-cell adhesion. CC analysis indicated that the targets were mainly associated with vesicle lumen, cytoplasmic vesicle, and membrane raft. MF analysis suggested that the targets were mainly involved in endopeptidase activity and serine-type endopeptidase activity, which indicated that the effect of baicalin to treat alopecia proceeds through these main processes (Figure 2(a)). KEGG pathway enrichment analysis was performed on the screened potential target proteins using the Metascape database, and a total of 93 important pathways were obtained, mainly involving proteoglycans in cancer signaling pathways, as well as the PI3K/AKT and forkhead box O signaling pathways. According to the *P* value, the top 20 pathways were selected to draw the corresponding bubble diagram (Figure 2(b)). The results suggested that the PI3K/AKT signaling pathway has an important role in alopecia.

### 3.3. Effect of Baicalin or LY294002 on the Behavior of HHDPCs

The dermal papilla is located at the base of the hair follicle, which generates hair fibers by inducing the development of epidermal hair follicles and has a vital role in the hair growth cycle.

**Figure 3:**
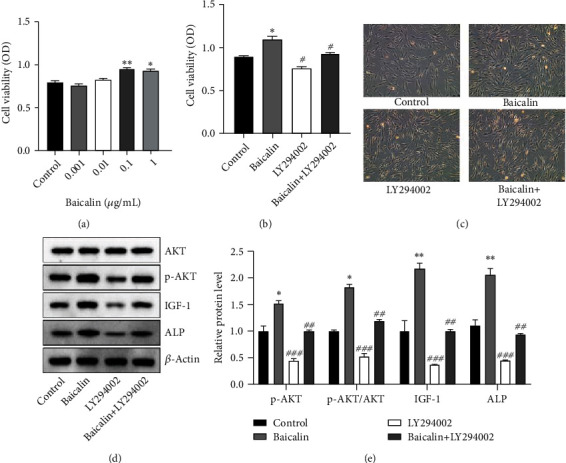
Effects of baicalin or LY294002 *in vitro*. (a) HHDPC viability following baicalin treatment. (b) Effect of baicalin or LY294002 on HHDPC viability. (c) Representative images of the different HHDPC groups (scale bar, 100 *μ*m). (d) Western blot for the detection of the protein levels of AKT, p-AKT, IGF1, and ALP and (e) quantified relative expression values/ratios. ^∗^*P* < 0.05 and ^∗∗^*P* < 0.01 vs. the control group; ^#^*P* < 0.05, ^##^*P* < 0.01, and ^###^*P* < 0.001 vs. the baicalin group. HHDPC: human hair dermal papilla cell; IGF1: insulin-like growth factor-1; ALP: alkaline phosphatase; p-AKT: phosphorylated AKT; OD: optical density.

Compared with the control group, 0.1 *μ*g/ml baicalin had the strongest effect on HHDPC proliferation (Figure 3(a)). In a parallel study, proliferation-induced HHDPCs were able to be blocked by PI3K inhibitor LY294002 (2 *μ*M, Figure 3(b)). As presented in Figure 3(c), the cell density was at its highest in the baicalin group. When compared with the baicalin group, the cell density decreased following treatment with baicalin plus LY294002. This observation suggests that LY294002 inhibited the baicalin-induced cell proliferation. As indicated in Figures 3(d) and 3(e), compared with those in the control group, the protein levels of p-AKT, IGF1, and ALP were increased, while LY294002 treatment reduced that upregulation.

### 3.4. Evaluation of the Hair Growth Cycle

**Figure 4:**
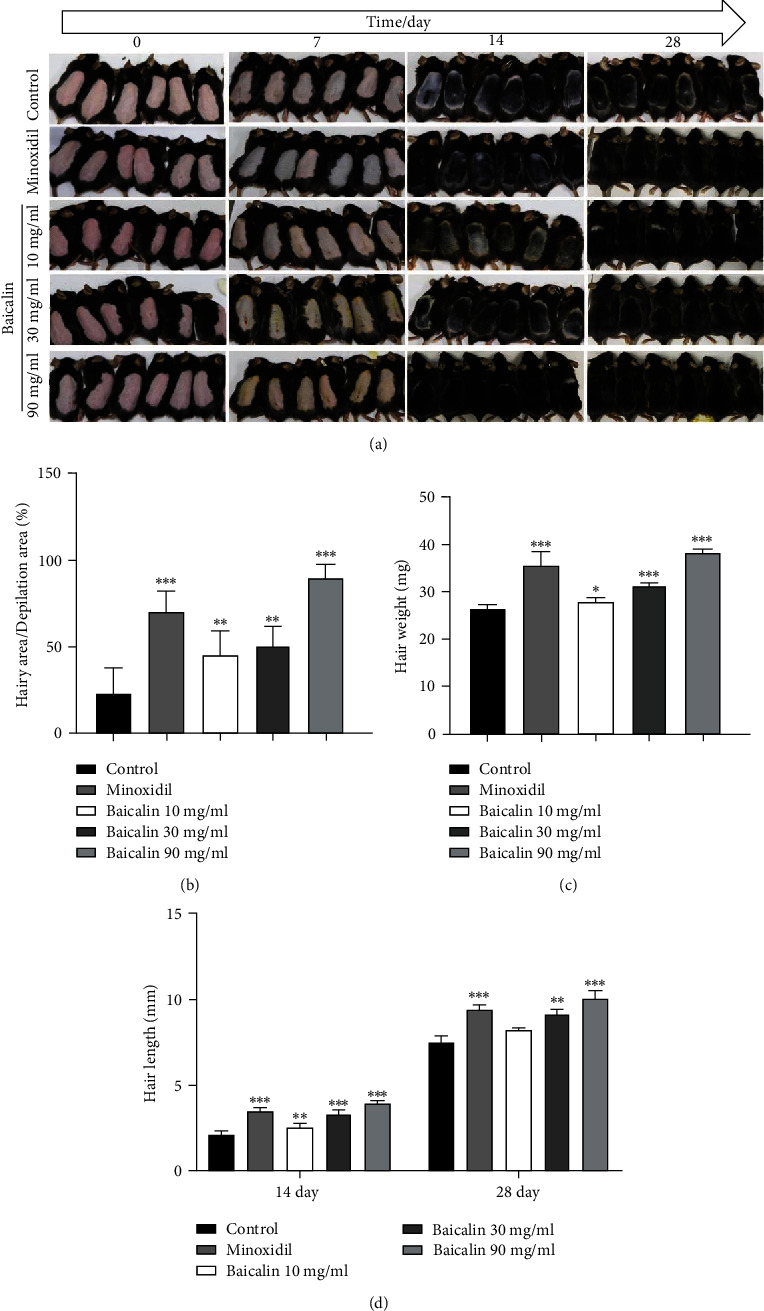
Effects of baicalin on hair regeneration in C57BL/6 mice. (a) Photographs were taken on days 0, 7, 14, and 28 days after applying baicalin on the shaved dorsal skin. (b) Hair area, (c) weight, and (d) length of mouse hair on day 14 and 28. ^∗^*P* < 0.05, ^∗∗^*P* < 0.01, and ^∗∗∗^*P* < 0.001 vs. control group.

In the display of the hair growth status of C57BL/6 mice, photographic images were captured at intervals of 7, 14, and 28 days. At 7 days after the treatment, the skin color of the mice changed from pink to light gray. On day 14, the epidermis of the mice in each group appeared gray and exhibited fine hairs. On day 28, the entire depilated area on the back of the mouse was covered with new hair (Figure 4(a)). Following baicalin treatment, the coverage and weight of the hair increased in a baicalin dose-dependent manner, and the effect of 90 mg/ml was better than that of the positive drug minoxidil (Figures 4(b) and 4(c)). On days 14 and 28, following baicalin treatment, the length of the hair had increased in a dose-dependent manner, and the effect of 90 mg/ml was almost the same as that of the positive drug minoxidil (Figure 4(d)).

### 3.5. Histological Observation of Hair Follicles

**Figure 5:**
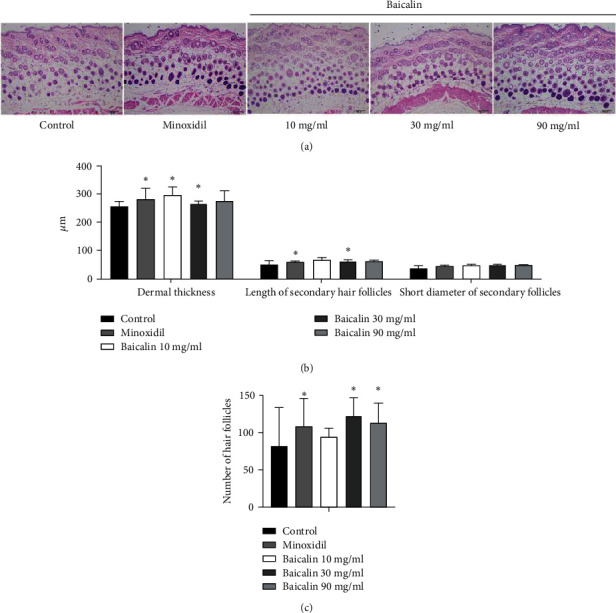
Effects of baicalin on hair follicles in C57BL/6 mice. (a) H&E staining of the skin in each group following minoxidil or baicalin treatment (scale bar, 100 *μ*m). (b) Hair follicle growth status in the different groups: dermal thickness, length of secondary hair follicles, and short diameter of secondary hair follicles. (c) Mean number of hair follicles from at least 6 replicates of the samples. ^∗^*P* < 0.05 vs. control group. H&E: hematoxylin and eosin.

H&E staining was performed on cross-sections of the skin to determine the effect of baicalin on hair follicle growth. In the control group, most hair follicles were in the telogen phase, with low numbers in the catagen phase and nearly no follicles in the anagen phase. The mice in the baicalin and 90 mg/ml minoxidil groups had larger hair follicles, the formation of melanin was obvious, the number of hair follicles was large, and the shape was complete (Figure 5(a)). The dermal thickness, length of secondary hair follicles, and short diameter of secondary follicles in the selected area were the smallest in control mice. As compared with the control group, both minoxidil and baicalin treatment promoted the increase of these three indicators (Figure 5(b)). The number of hair follicles in six different areas was observed under an optical microscope at a magnification of x100 (Figure 5(c)), and the 30 and 90 mg/mL of baicalin treatment groups have more hair follicles than the control group.

### 3.6. Detection of Key Proteins from Bioinformatics Network

**Figure 6:**
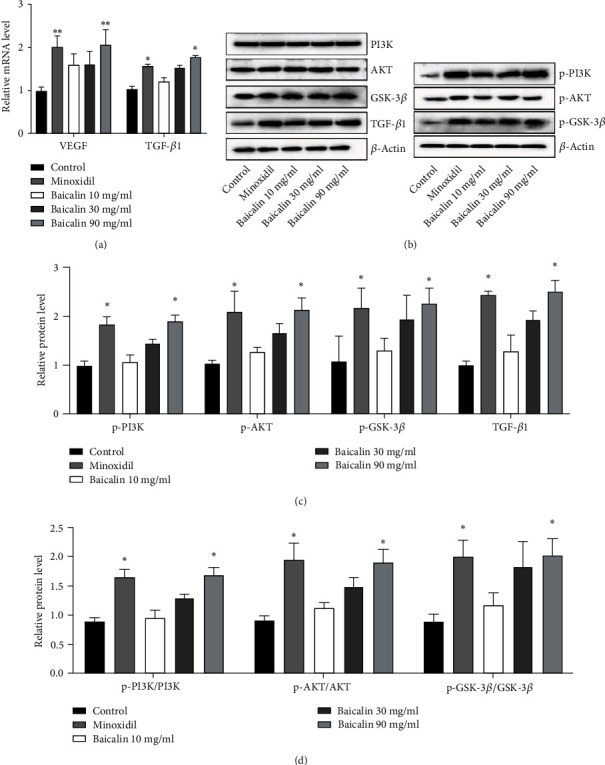
Influence of baicalin on hair growth-related genes. (a) Relative mRNA level of VEGF. (b) Relative protein expression of PI3K, p-PI3K, AKT and p-AKT, GSK-3*β*, p-GSK-3*β,* and TGF-*β*1 (target protein-OD/*β*-actin-OD). (c) Relative protein expression p-PI3K, p-AKT, p-GSK-3*β*, and TGF-*β*1 (phosphorylated protein-OD/total protein-OD). (d) Phosphorylated proteins/total protein levels for PI3K, AKT, and GSK-3*β*. ^∗^*P* < 0.05 and ^∗∗^*P* < 0.01 vs. the control group. VEGF: vascular endothelial growth factor; GSK: glycogen synthase kinase; p-AKT: phosphorylated AKT; OD: optical density.

Skin tissues from mice were used to detect the effects of baicalin on the mRNA expression of TGF-*β*1 and VEGF. In addition, the expression of key proteins [PI3K, p-PI3K, AKT, p-AKT, glycogen synthase kinase (GSK)-3*β*, p-GSK-3*β,* and TGF-*β*1] was detected using western blot analysis. As compared with the control group, the positive drug minoxidil induced the mRNA expression of VEGF and TGF-*β*1 (Figure 6(a)). Concerning PI3K, p-PI3K, AKT, p-AKT, GSK-3*β*, p-GSK-3*β,* and TGF-*β*1 proteins, both minoxidil and baicalin treatment significantly induced the phosphorylation of PI3K, AKT, and GSK-3*β* and induced TGF-*β*1 compared with the control group, and baicalin had a similar effect to that of minoxidil (Figures 6(b) and 6(c)). According to the ratio of phosphorylated proteins PI3K, AKT, and GSK-3*β* to total proteins in Figure 6(d), minoxidil and baicalin had no significant effect on these total proteins but were able to induce enhanced phosphorylation of these three proteins.

## 4. Discussion

As an important appendage of skin organs, hair is a unique feature of mammals [18, 19]. The main functions of hair include protecting the skin from mechanical damage, heat preservation, and sensory functions, and it has an influence on social interaction, psychology, and quality of life [20, 21]. According to statistics, the number of individuals with hair loss in China has reached 250 million, and the proportion of those people that are aged <30 has been increasing annually, with a younger trend observed globally [20–23]. However, the availability of novel drugs for the treatment of alopecia has not markedly increased. Baicalin, as an active monomer of TCM, has been proven to promote the proliferation of HHDPCs *in vitro* [6]. *Scutellaria baicalensis* Georgi extract has been reported to affect the function of the liver, and its active compound, baicalin, was indicated to inhibit androgen receptor translocation, which is important for the prevention of androgenetic alopecia [24–26]. In the present study, baicalin was observed to promote hair growth in C57BL/6 alopecia areata model mice, and baicalin promoted HHDPC proliferation by activating the AKT/IGF1 signaling pathway and increasing the activity of ALP.

In the present study, networks pharmacology was used to assist in exploring the potential mechanism of action of baicalin in the treatment of hair loss. Networks pharmacology integrates systems biology, networks biology, computational biology, multidirectional pharmacology, molecular pharmacology, molecular dynamics, and other multidisciplinary technologies and content [27, 28]. Drug screening may be performed through systems biology and network analysis, including network construction and visualization, network topology attribute analysis, and physical function analysis [29]. The present study predicted 189 potential targets of baicalin for the treatment of alopecia, with 122 overlapping targets with alopecia, indicating that baicalin has multiple targets in the treatment of alopecia. The networks pharmacology analysis results suggested that the functions of baicalin were significantly enriched in the regulation of neutrophil degranulation, regulation of cell-cell adhesion, vesicle lumen, cytoplasmic vesicle, membrane raft, and endopeptidase activity, mainly through their effects on AKT1, Src protein, EGFR, and IGF1.

DPCs are located at the base of hair follicles and have an important role in regulating the periodic circulation of hair follicles and promoting hair growth [30]. By secreting numerous biologically active factors, they form a signaling molecule network to promote the proliferation and differentiation of cells in the hair follicle, thereby promoting hair growth [31]. In the present study, it was observed that 0.1 *μ*g/ml baicalin had a significant effect on HHDPC proliferation. Baicalin at a concentration of 0.1 *μ*g/ml was able to induce the phosphorylation of AKT and activate IGF1 and ALP. This effect was blocked by the inhibitor LY294002, indicating that the PI3K/AKT pathway acts as a signaling pathway in alopecia, which is consistent with the results of the networks pharmacology analysis. A previous study indicated that bacillus/*Trapa japonica* fruit extract ferment filtrate enhances human hair follicle dermal papilla cell proliferation via the AKT/ERK/GSK-3*β* signaling pathway [32]. IGF1 is similar in structure to insulin and may stimulate the proliferation of melanocytes and epithelial cells in hair follicles [33]. ALP expression fluctuates with the hair growth cycle; it is the strongest in the early growth period and begins to decline in the late growth period [31]. The activation of AKT signaling by baicalin and increased activation of IGF1 and ALP in HHDDCs may be a potential mechanism of hair growth promotion.

In the present study, C57BL/6J mice were selected as a model to study the utility of baicalin in the treatment of alopecia. The hair of C57BL/6J mice grows cyclically, and the hair cycle is divided into the growth, regression, and resting phases [34]. Melanocytes in the skin are distributed to the hair follicles [35]. Synthesis, secretion, and darkening of melanin occur during hair growth [36]. The hormone is delivered to the keratinocytes, making the skin look black. During the hair regression period, melanin synthesis decreases and the skin gradually changes from black to gray.

Currently, mouse dorsal skin depilation is a common method to study alopecia [16, 17]. There is no melanin synthesis during the resting period and the skin is pink, so the state of the hair growth cycle may be identified by the skin color of C57BL/6J mice [16]. In the present study, hair removal cream was used to remove the hair on the back of C57BL/6J mice, so that the hair growth was in the resting phase, and an alopecia model was thus established, causing minimal damage to the experimental animals. This study replicates the hair removal mice based on the studies of Deng et al. [16] and Zhang et al. [17], a model that may be used to study the effects of drugs on the onset of hair growth. The results indicated that the skin color of the alopecia area in the baicalin group became darker after 7 days of treatment. After 28 days of baicalin treatment, mice in the baicalin group had shinier and longer hair as well as a larger hair coverage area. The effects of baicalin treatment were comparable to those of minoxidil treatment. The longest hair length in the baicalin-treated group indicated that the HHDPCs were in an activated state and were able to provide sufficient nutrients for hair growth.

For the hair cycle, the number and shape of hair follicle precursor cells, as well as their proliferation rate, affect the length and fineness of the hair; the longer the hair follicle growth period, the longer the hair [18]. The histopathological sections revealed that there were more hair follicles following baicalin treatment, the hair follicles were long and large, and the expression of melanin was obvious. TGF-*β*1, VEGF, PI3K, AKT, and GSK-3*β* have been reported to be involved in the occurrence and development of alopecia. TGF-*β*1 is a multifunctional growth factor that has multiple effects on the development of skin hair follicles [37]. It is able to regulate cell proliferation, differentiation, and apoptosis [38]. It has an important role in inducing the anagen phase of the hair cycle. VEGF may regulate the periodic circulation of hair follicles and promote hair growth by inducing the proliferation and migration of DPCs or inducing the formation of blood vessels around the hair follicles [39]. Vanillic acid was reported to stimulate the anagen phase by activating the PI3K/AKT/Wnt/*β*-catenin pathway and potentially alleviating hair loss [40]. The activation of GSK-3*β* may enhance the expression of the Wnt inhibitor Dickkopf-1 and catagen inducer TGF-*β*2 to induce hair follicle growth [41]. GSK-3*β* is an evolutionarily conserved serine/threonine kinase, and GSK-3*β* also acts on numerous signaling structural proteins and transcription factors. Previous studies have indicated that minoxidil promotes TGF-*β*1 to promote hair growth [42]. In the present study, VEGF and TGF-*β*1 expression as well as the activating phosphorylation of PI3K, AKT, and GSK-3*β* were induced by baicalin to varying degrees, and this response may have been accountable for the positive effect on hair growth. The regulatory function of GSK-3*β* signaling is dependent on the nuclear transcription of *β*-catenin. Therefore, the nuclear translocation of *β*-catenin will be the focus of the next study.

In conclusion, in the present study, the main targets of baicalin in the treatment of alopecia were studied using networks pharmacology, and the interaction of antialopecia targets was predicted. The BP and pathway enrichment of the intersecting targets were elucidated. Multiple pathways of the antialopecia effects provided a scientific basis for experimental research and pharmaceutical product development based on baicalin's antialopecia mechanism. The biological experiments proved that baicalin is able to promote hair growth, as it may induce mouse hair to undergo the growth phase early, promote the proliferation of HHDPCs, and upregulate/activate AKT, IGF1, and ALP in HHDPCs cells, thereby promoting hair growth. As compared with minoxidil, baicalin is sourced naturally and mild, which has broadened the prospects for its development and application. However, several issues remain in the application of networks pharmacology, such as perfecting and optimizing the biological information database of TCM and constructing a dynamic spatial network model. Furthermore, it remains to be explored whether there are further ways to promote baicalin-induced hair growth. In addition, subsequent *in vivo* experiments with more mice in each group may be performed to further study the effects of baicalin on alopecia.

## Figures and Tables

**Table 1:** Primer sequences used for PCR.

Gene	Direction	Sequence (5′-3′)
VEGF	Forward	AGTCTGTGCTCTGGGATTTGAT
	Reverse	GCTCTTGATACCTCTTTCGTCTG
TGF-*β*1	Forward	GGAGCCCGAAGCGGACTA
	Reverse	GCGTTGTTGCGGTCCAC
*β*-Actin	Forward	CACGATGGAGGGGCCGGACTCATC
	Reverse	TAAAGACCTCTATGCCAACACAG

## Data Availability

The data generated in the present study may be requested from the corresponding author upon reasonable request.

## Abbreviations

HHDPCs: Human hair dermal papilla cells
EGFR: Epidermal growth factor receptor
IGF1: Insulin-like growth factor 1
AKT1: Protein serine/threonine kinase
TCM: Traditional Chinese medicine
TNF-*α*: Tumor necrosis factor *α*
MF: Molecular function
CC: Cellular component
BP: Biological process
KEGG: Kyoto Encyclopedia of Genes and Genomes
NF-*κ*B: Nuclear factor kappa-B
TGF-*β*1: Transforming growth factor-*β*1
GSK-3*β*: Glycogen synthase kinase-3*β*.
